# Rutile Ru_x_Ti_1-x_O_2_ nanobelts to enhance visible light photocatalytic activity

**DOI:** 10.1038/s41598-019-55446-7

**Published:** 2019-12-11

**Authors:** S. Mihai, D. L. Cursaru, D. Matei, A. M. Manta, R. Somoghi, G. Branoiu

**Affiliations:** 1 Petroleum – Gas University of Ploiesti, 39 Bucharest Av., 100680 Ploiesti, Romania; 2 National Research and Development Institute for Chemistry and Petrochemistry, 202 Splaiul Independentei, 060021 Bucharest, Romania

**Keywords:** Pollution remediation, Photocatalysis

## Abstract

We herein report on the synthesis by a facile sol-gel method without templates for preparing rutile Ru_x_Ti_1-x_O_2_ (x = 0.16; 0.07; 0.01) nanobelts with exposed (001) facets. The rutile nanobelts with exposure (001) facets, favor the separation photogenerated electron-hole pairs and inhibit the recombination of the electron-hole pairs resulting in the increase of the number of main superoxide and hydroxyl radicals. The photocatalytic properties of the rutile Ru_x_Ti_1-x_O_2_ nanobelts were evaluated by discoloring of MB (methylene blue) dye under sunlight irradiation at an intensity of 40000 lx. It was also done a thorough interface analysis to determine the band energy.

## Introduction

Titanium dioxide (TiO_2_) has been extensively investigated due to its chemical stability, low cost, catalytic properties, photocatalytic properties, environmental clean-up by organic compound mineralization^1^ and because it is a renewable energy generator used for efficient hydrogen production^2–4^.

TiO_2_ has four crystalline polymorphs, anatase, rutile, brookite and TiO_2_ (B) (biphasic based on anatase). Anatase and anatase biphasic TiO_2_ have been extensively investigated in photocatalysis^5^ under various forms and morphologies: microspheres, nanoflows, nanotrees and nanobelts^6–8^.

One of the objectives of photocatalysis is the efficient use and conversion of solar light, this is done by doping classical photocatalysts with noble metals (Au, Ag, Pt, etc.)^7,9,10^ or other oxides (RuO_2_)^11,12^. Also, understanding the principles of crystal growth is a major challenge for many researchers, surface science is an intensely studied field. In the last decade, the science of anatastic surface has attracted attention due to the special properties of the surface, the differences in reactivity and surface energy^13^. Yang to al.^13^ were the promoters of the TiO_2_ anatase crystal synthesis with a high percentage (47% by weight) of facets (001), using hydrogen fluoride. Nanobelts structures are considered 1D nanostructures. The dimensional study plays an important role in determining material properties and is a huge challenge for researchers. 1D nanostructures have been extensively investigated due to their distinctive properties related to 0D and 2D materials. 1D nanostructures show two important properties: fast electronic transport and effective load transfer^14^. Synthesis of 1D nanoparticles depends on several factors: chemical method, *p*H and temperature, while the catalytic activity depends mainly on the properties of the surface^8^. The increased interest in the controlled synthesis of the exposed face exposed TiO_2_ particles (001) is due to a high photocatalytic activity compared to (101) faces^15^. The study research of Zhao *et al*. have shown that surface energy of rutile TiO_2_ facets is γ (001) > γ (100) > γ (101) (0,90 J m-2 > 0,53 J m-2 > 0,44 J m-2)^14^. The chemical dissociation of the water molecules is energetically favored on the plane (001), and the hydroxyl radicals on the TiO_2_ surface react much more easily with the dissociated molecules of the organic compounds^14^.

In this paper, it was developed a facile sol-gel method without templates for preparing rutile.

Ru_x_Ti_1-x_O_2_ nanobelts crystals with controlled morphologies. The nanobelts have a layer structure which is beneficial for the introduction of heteroatoms, as an example Ru, because RuO_2_ adopts the rutile structure. Introduction of the metal ions into the crystalline structure of TiO_2_ nanobelts, expands the absorption edge to the visible light range to enhance, therefore we propose the introduction of ruthenium into the crystal for the extend of the absorption to the visible light range.

## Results

**Figure 1:**
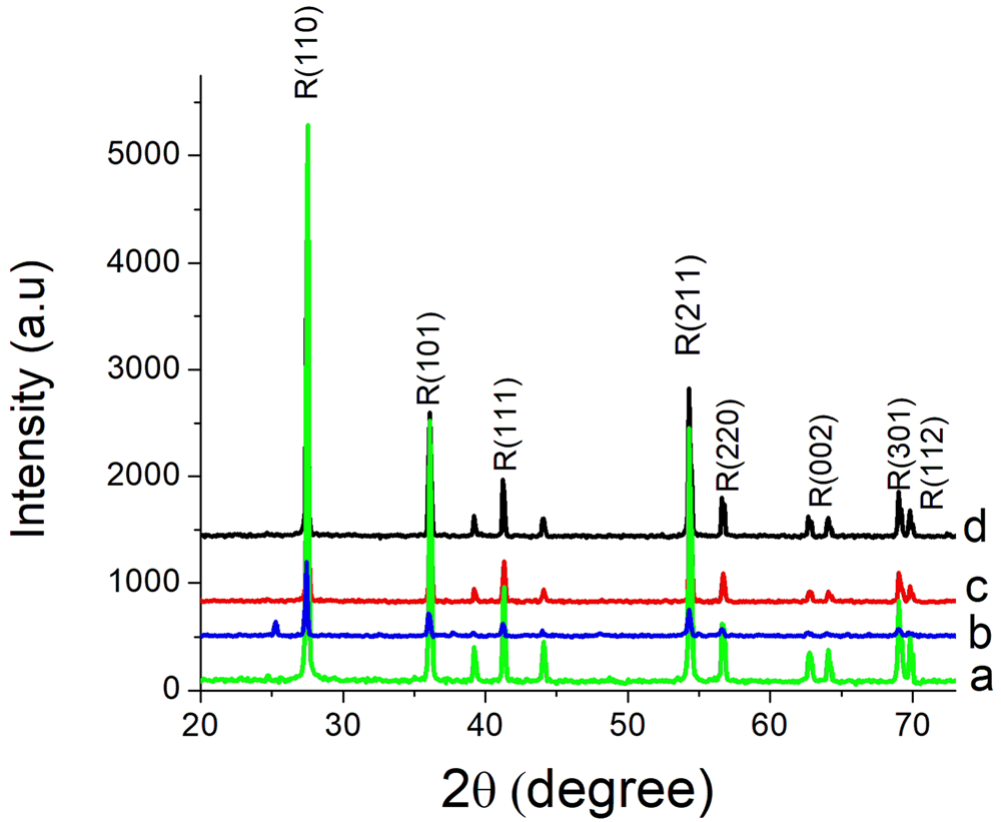
XRD patterns of Ru_x_Ti_1-x_O_2_ nanobelts a(x = 0); b(x = 0.01); c(x = 0.07); d(x = 0.16).

The crystalline phases structures of rutile TiO_2_ and rutile Ru_x_Ti_1-x_O_2_ nanobelts are shown in Fig. 1. RuO_2_ shows a rutile structure and it has lattice parameters similar to those of TiO_2_ rutile, therefore no new peaks were observed in the diffraction spectrums of nanobelts structures, regardless of Ru doping concentration.

Whatever of the Ru doping concentration, the typical diffraction peaks which appear at 2θ = 28°, 36°, 42°, 55°, 57°, 63°, 64°, 68°, 69°could be assigned as the (110); (101); (111); (211); (220); (002); (310); (301); (112) diffraction lines corresponding to the rutile phase. The crystalline phase composition and lattice parameters from rutile nanobelts, having a tetragonal phase with lattice parameters a = b from 4.587 to 4.595 Å, and c = 2.957 to 2.967 Å is obtained with very high crystalline quality. The crystalline phase composition and lattice parameters from Rietveld refinement are summarized in Table 1.

**Table 1:** The crystalline phase composition and lattice parameters from Rietveld refinement.

Sample	Space group	Z	Lattice parameters (Å)		d_110_ (Å)	Cell Volume (Å^3^)	Crystal Density (g/cm^3^)
			a (=b)	c			
Ti_1-x_Ru_x_O_2_, x = 0.01	P42/mnm	2	4.587	2.957	3.243	62.240	4.314
Ti_1-x_Ru_x_O_2_, x = 0.07	P42/mnm	2	4.592	2.959	3.247	62.466	4.463
Ti_1-x_Ru_x_O_2_, x = 0.16	P42/mnm	2	4.595	2.967	3.250	62.474	4.648

**Figure 2:**
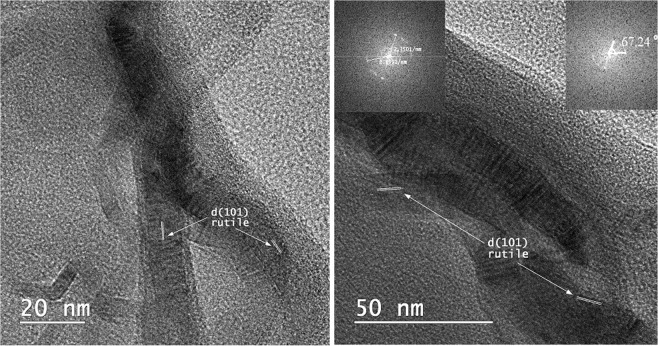
HRTEM images for Ru_x_Ti_1-x_O_2_ (x = 0.16) nanobelts.

It should be noted that the larger lattice constants and d-spacing values are due to upon Ru doping. This is due to the larger ionic radius of Ru than that of Ti in the rutile, the structural relaxation follows the Vegard’s law^16^. The d-spacing value of the rutile sample for (110) plane is in agreement to the interatomic dimension observed at HRTEM microscopy. Figure 2 shows the HRTEM images of Ru_x_Ti_1-x_O_2_ (x = 0.16) nanobelts sample. The TEM images confirm the formation of one-dimensional nanobelts nanostructures. Rutile TiO_2_ nanobelts have very small thicknesses, widths of 6–20 nm and can be 100–200 nm in length.

The HRTEM image shows the (101) and (002) atomic planes with lattice spacings of 3.499 Å (1/r) and 4.255 Å (1/r), respectively^17^. The interfacial angle between these two crystalline facets was found to be 67.27° and it was determined by Fast-Fourier Transform (FFT) image (Fig. 2). Deviation with one degree may be due to ruthenium from the crystalline lattice. These results even if less than the theoretical value, reveal that the interfacial angle between rutile is in good agreement with the theoretical value of the angle between the (101) and (001) planes. HRTEM images suggest that the prepared sample behaved like a well-crystallized heterostructure nanobelts.

Rutile Ru_x_Ti_1-x_O_2_ nanobelts have an octahedral arrangement, where either Ti or Ru atoms prefer a coordination number of 6. The layered arrangements of octahedrons facilitate their growth in the (001) direction as nanobelt like geometry^14^.

**Figure 3:**
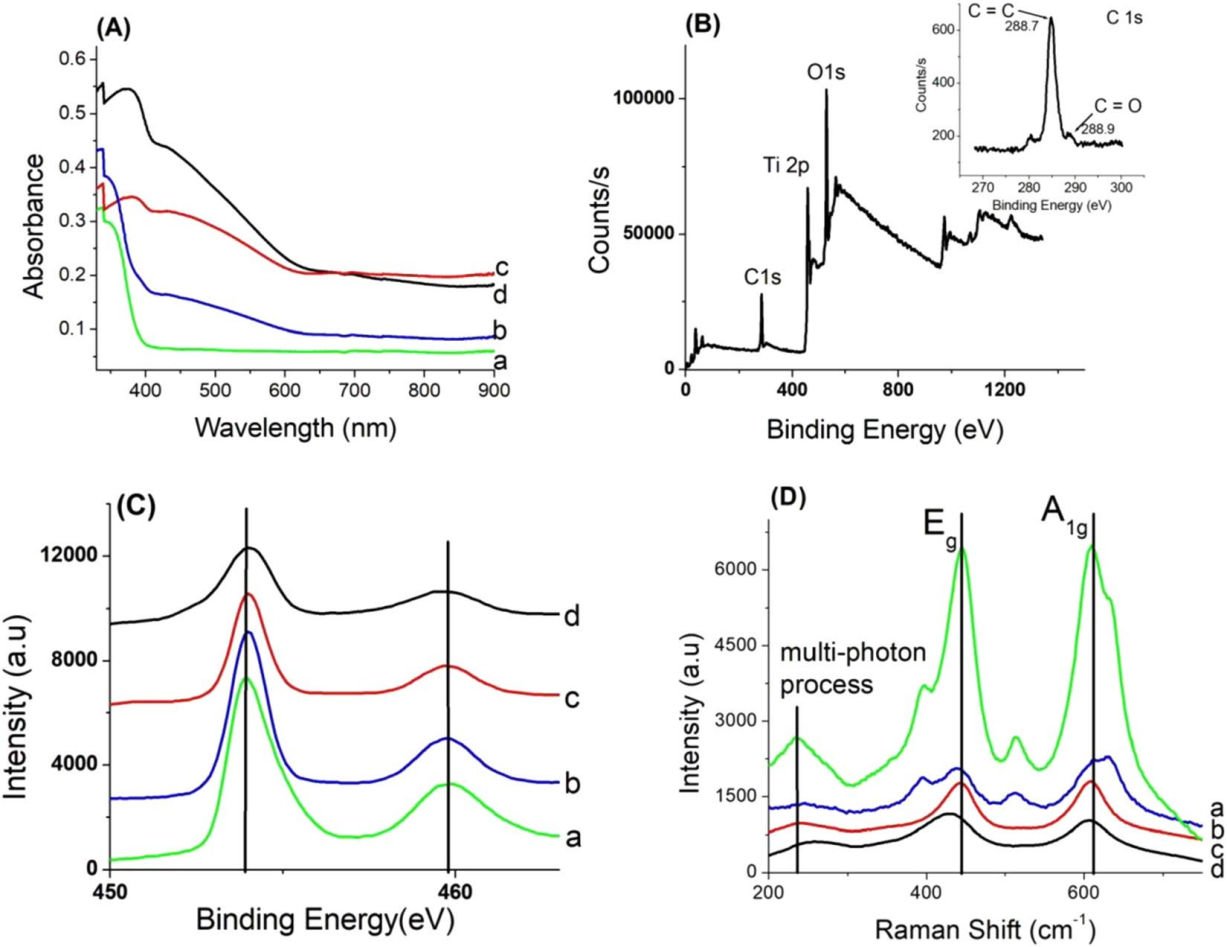
(**A**) UV-vis spectra of Ru_x_Ti_1-x_O_2_ nanobelts; (**B**) XPS survey spectrum of Ru_x_Ti_1-x_O_2_ (x = 0.16) nanobelts; (**C**) High resolution core XPS spectra of Ru_x_Ti_1-x_O_2_ nanobelts a(x = 0); b(x = 0.01); c(x = 0.07); d(x = 0.16); (**D**) Raman spectrum of Ru_x_Ti_1-x_O_2_ nanobelts.

The UV-Vis diffuse reflectance spectroscopy (DRS) spectra of all samples are shown in Fig. 3A. The DRS method is employed to determine the band gap energy for photocatalysts. The intense absorption feature in the range of 200 nm–408 nm is characteristic of the TiO_2_ and corresponds to the band gap energy of 3.2 eV as for rutile phase. The visible absorption band in the range 408–627 nm corresponds to the band gap energy from 2 to 3.2 eV and can be assigned to charge transfer transition of the donor (Ru^4+^ → Ru^5+^  + e^−^, Ru^3+^ → Ru^4+^  + e^−^) or acceptor (Ru^4+^ → Ru^3+^  + h^+^) type^18,19^.

**Figure 4:**
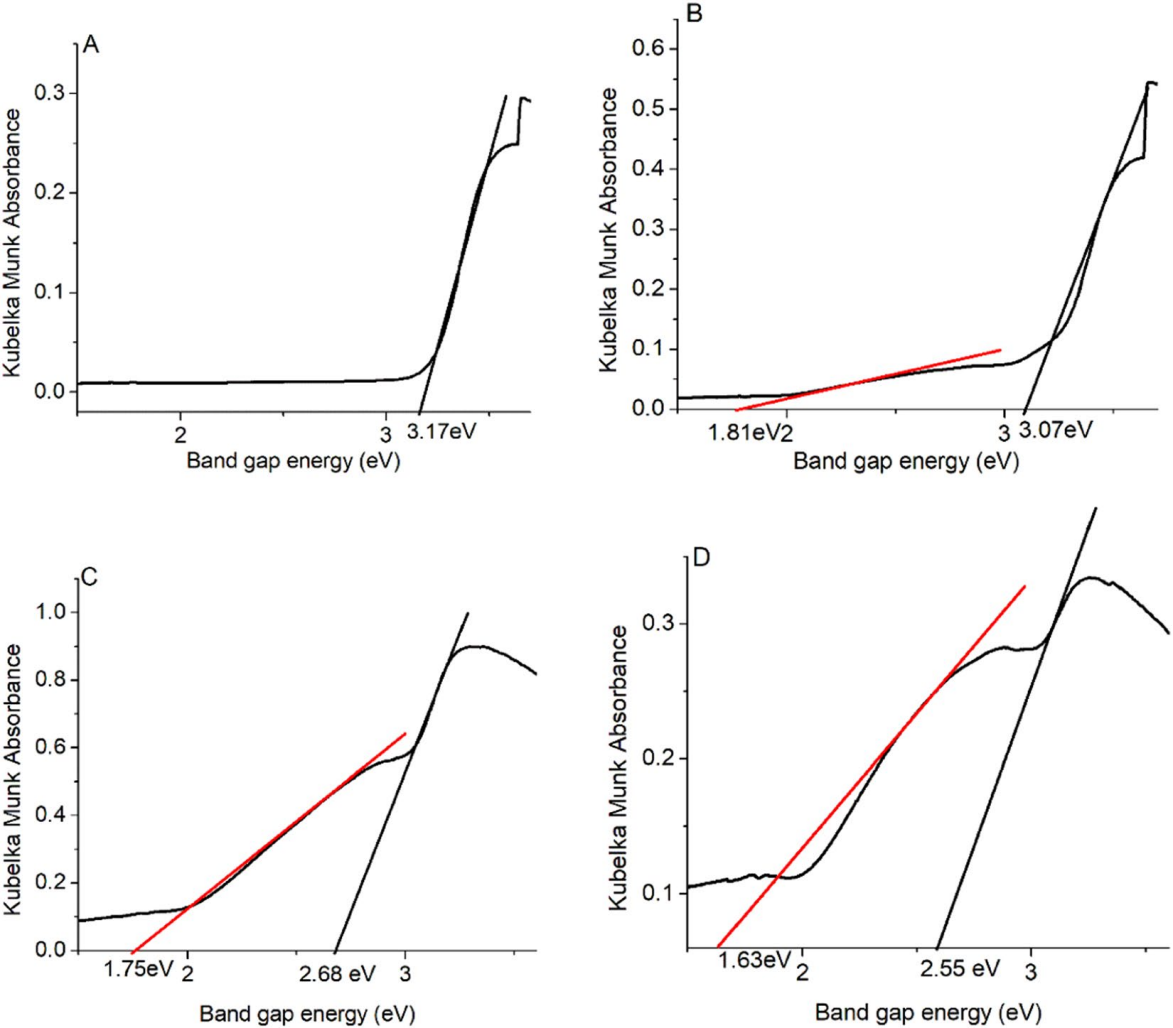
Determination of energy band gap of Ru_x_Ti_1-x_O_2_ nanobelts a (x = 0); b (x = 0.01); c (x = 0.07); d (x = 0.16).

This suggests that rutile Ru_x_Ti_1-x_O_2_ photocatalysts could be active in the visible light region. The specters UV-vis from rutile Ru_x_Ti_1-x_O_2_ photocatalysts were transformed to the absorption specters according to the Kubelka Munk theory: ${[{\boldsymbol{F}}({{\boldsymbol{R}}}_{\infty })\cdot {\boldsymbol{hv}}]}^{1/2}$ (Fig. 4). The optical band gap energies of the photocatalysts can be approximated from the plot of ${[{\boldsymbol{F}}({{\boldsymbol{R}}}_{\infty })\cdot {\boldsymbol{hv}}]}^{1/2}$ versus hν (photon energy) and were estimated from the intercept of the tangent with the abscissa axis yielding the band gap energies. It can be observed that the gap energies band is presented in Fig. 4 compared to the bulk rutile TiO_2_ of 3.2 eV. The bandgap energies decreased from 3.2 eV for rutile TiO_2_ to 2.55 eV, 2.68 eV 3.07 eV for Ru_x_Ti_1-x_O_2_ x = 0.16, x = 0.07 and x = 0,01 respectively.

XPS measurements have been performed to analyze the surface composition and oxidation states of Ti and Ru in the samples. The survey spectrum for the Ru_x_Ti_1-x_O_2_ (x = 0.16), with the highest ruthenium content (Fig. 3B), confirms the complete removal of chlorine in the samples TiO_2_ doped. The absence of the peaks in area 197.9 eV and 199.5 eV (Cl 2p3/2 and Cl 2p1/2) were observed. Figure 3C shows the XPS spectra of Ti 2p on the surface of each sample. The binding energies at 454.0 and 459.7 eV (Ti 2p3/2 and Ti 2p1/2) correspond to octahedral coordinated Ti^4+^ state^19,20^. The energy difference between the two peaks is 5,7 eV which is consistent with the energy difference between the level of spin-orbit splitting coupling effect. c (Fig. 3C). The substitution of Ru ions into the TiO_2_ lattice can induce electronic structure.

The Raman spectra for rutile Ru_x_Ti_1-x_O_2_ photocatalysts are displayed in Fig. 3D, where two bands features of tetragonal rutile TiO_2_ (space group D_4h_) at 445 and 610 cm^−1^ were assigned to E_g_ (planar O-O vibration) and A_1g_ (Ti-O stretch) modes 20. The broad band at 235 cm^−1^ was attributed to the multiple photon scattering process. The absence of the peaks to features attributable to RuO_2_ from 528 cm^−1^ and 646 cm^−1^ corresponds to E_g_ and A_1g_ modes^21^, was evident in all of the samples, confirming the lattice substitution of the RuO_2_ in the rutile TiO_2_. Ruthenium doping induces a low red-shifts with increasing Ru concentration in all of the samples, probably due to the formation of oxygen defects or Ru-O-Ti linkages (Fig. 3D).

**Figure 5:**
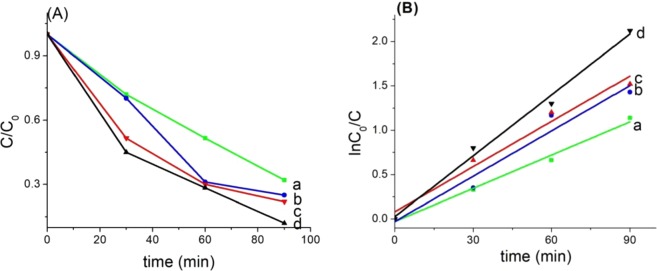
(**A**) The C/C_0_ versus time curves of photocatalytic degradation of MB; (**B**) Photocatalytic performances of Ru_x_Ti_1-x_O_2_ nanobelts a(x = 0); b(x = 0.01); c(x = 0.07); d(x = 0.16).

The photocatalytic properties of the rutile Ru_x_Ti_1-x_O_2_ nanobelts have been evaluated by photocatalytic degradation of methylene blue (MB) under sunlight at the 40000 lx intensity. The photocatalytic results are displayed in Fig. 5A. It was observed that the photodegradation process of methylene blue took place faster in the presence of rutile doped with ruthenium compared with undoped rutile. The increase of ruthenium content gave a decrease of the band gap that can be correlated with the increase absorption to the visible light range, and respectively enhance of the photocatalytic activity. It was observed that the photodegradation process of methylene blue took place faster in the presence of rutile Ru_x_Ti_1-x_O_2_ (x = 0.16) nanobelts compared to the other synthesized photocatalysts. It can be observed that after irradiation for 90 min, about 90% of the methylene blue has been degraded for the Ru_0.16_Ti_0.84_O_2_ nanobelts which are above the results of the rutile TiO_2_ (74 wt.%).

The kinetic curves of sunlight MB degradation over each catalyst are depicted in Fig. 5B and show that the degradation of MB dye follows pseudo first-order kinetics law, lnC/C_0_ = k_app_t, where k_app_ is the pseudo-first order constant rate.

The constant rate k_app_, was determined by plotting the lnC/C_0_ versus irradiation time. It can be observed that with increasing of the ruthenium content the constant rate k_app_ rises from 0.0125 min^−1^ to 0.0227 min^−1^ (Table 2).

**Table 2:** The rate constant k_app_ of Ru_x_Ti_1-x_O_2_ nanobelts.

k_app_	Ru_x_Ti_1-x_O_2_ (x = 0.16)	Ru_x_Ti_1-x_O_2_ (x = 0.07)	Ru_x_Ti_1-x_O_2_ (x = 0.01)	TiO_2_
k_app_ MB (min^−1^)	0.02273	0.0170	0.0168	0.0125

**Figure 6:**
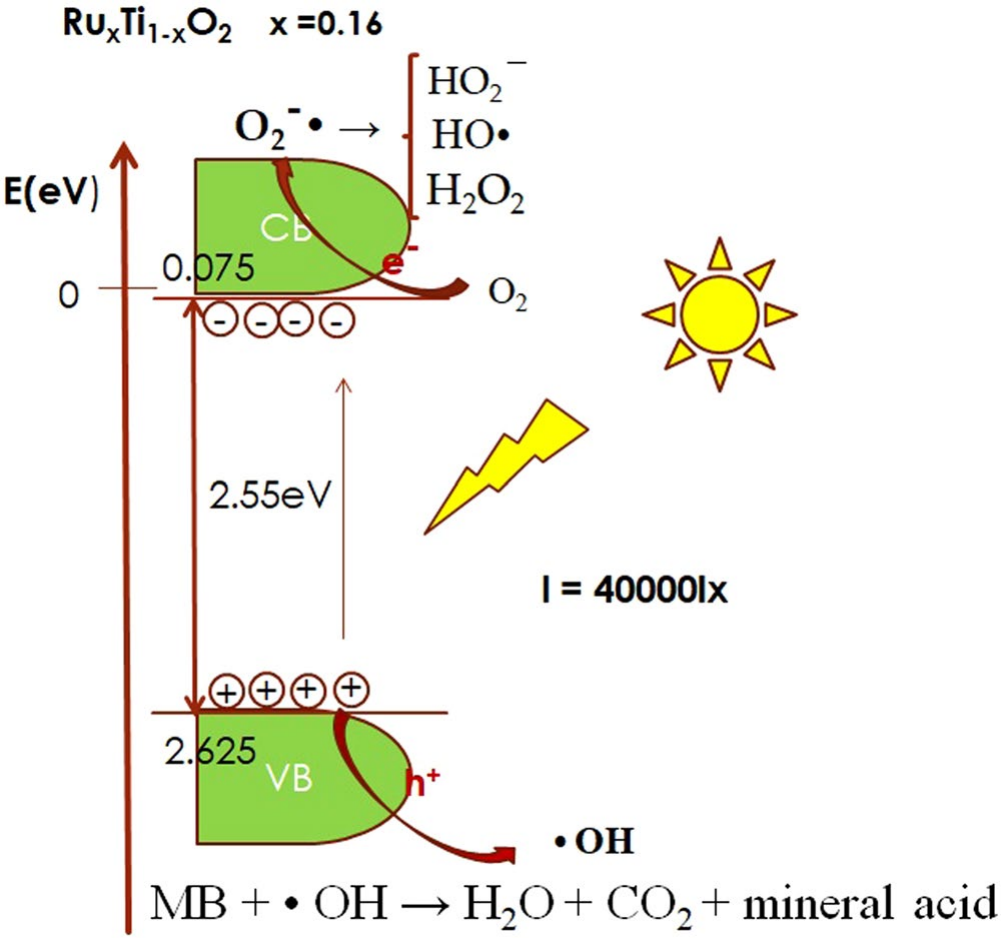
Mechanism of Ru_x_Ti_1-x_O_2_ nanobelts photocatalysis.

On the basis of the experimental results, a possible mechanism of the enhanced photocatalytic activity over the rutile Ru_x_Ti_1-x_O_2_ is proposed in Fig. 6.

The enhanced photocatalytic activity caused by ruthenium doping and to the ability of Ru to capture the photogenerated holes on the TiO_2_ (valence band). The conduction band can be calculated by using the empirical equation^22^:

Ecb = Evb − Eg and Evb of semiconductor were estimated by the following equation:

Evb = X − Ee + 1/2Eg, where Ecb is the conduction band, Evb is the valence band, X the geometric mean of the Mulliken, Ee is the energy of free electrons on the hydrogen scale (~4.5 eV) and Eg is the bandgap value of semiconductor, respectively. The values of Ecb, Evb are displayed in Table 3.

**Table 3:** The values Ecb, Evb of Ru_x_Ti_1-x_O_2_ nanobelts.

Energy (eV)	Ru_x_Ti_1-x_O_2_ (x = 0.16)	Ru_x_Ti_1-x_O_2_ (x = 0.07)	Ru_x_Ti_1-x_O_2_ (x = 0.01)	TiO_2_
E_g_	2.55	2.68	3.07	3.17
X	5.85	5.83	5.81	5.80
E_CB_	0.075	−0.01	−0.225	−0.285
E_VB_	2.625	2.67	2.845	2.885

It can be observed that hydrogen peroxide and peroxide radicals can form. The photocatalytic process is shown in Fig. 6. Excitaded electrons under sunlight jumped from VB (valence band) to CB (conduction band) and generate charge carriers (electron-hole pairs). Due to the nanobelts structure, the electron-hole pairs are moved to the photocatalyst surface, where electrons and holes are involved in redox reactions on the surface. The photocatalytic degradation efficiency can be due to the reactive (001) facets, these having a strong ability to dissociate water molecules to form hydrogen peroxide and peroxide radicals, contributors in the photo-oxidation process^16^.

## Discussions

Ru_x_Ti_1-x_O_2_ nanobelts were synthesized by the sol-gel method by doping TiO_2_ with RuO_2_. The reaction was carried out in HCl medium (c > 10 mol/l), which facilitated the formation of nanobelts nanostructures. RuO_2_ has been chosen for doping since both RuO_2_ and TiO_2_ have the same crystalline crystal structure, the crystalline parameters have almost equal values, coordinate number 6 for both Ti and Ru. From the diffraction spectrum, the rutile phase is observed for all nanobelts with high crystallinity. Also, doping TiO_2_ with ruthenium has a double role: decrease the bandgap and facilitating light from the visible field in the photocatalysis process, as well as to inhibit the recombination of the electron-hole pairs.

UV-vis absorption spectra were transformed using the Kubelka Munk function and the values of the bandgap energy for all samples with which the values of conduction bands and valence bands were calculated. In both the UV-vis and the transformed Kubelka Munk spectra, there are two types of transitions: interband between the 2p orbitals of the oxygen and the orbitals of the ruthenium and an interband type d-d between the orbitals of Ru (1.65–1.81 eV)^23–25^. TiO_2_ is considered a n-type semiconductor. There is a decrease in the bandgap from 3.17 to 2.55 eV as the content of Ru concentration increases in the samples, there is also an increase in the conductive band value, respectively, a decrease of valence value of nanobelts as the increase in doping with Ru. This favors the splitting of water and the formation of oxidoreduction species. Corroborated with the shape of nanostructures with surface exposure (001) facets showed the performance of efficient photocatalysts in the degradation of organic compounds. Doping with Ru induces a stress in the crystal of Ru_x_Ti_1-x_O_2_, which is revealed by an angle deviation of 68.3° to 67.3° for the (001) facets. Crystal stress is also noticeable in Raman spectra by easily moving to redshifts the bands assigned to Eg and A1g corresponding to the rutile phase of TiO_2_. The XPS spectrum shows a slight (low) displacement of the corresponding Ti 2p_3/2_ and Ti 2p_1/2_ blue-shifts spectra due to ruthenium doping. The high performance of the photocatalysts is due both to the nanobelts form and to the ruthenium doping and the visible light access to the photodegradation process. The superoxide and hydroxyl radicals obtained on the photocatalyst surface favor the mineralization reaction of the organic dye by in CO_2_ and H_2_O.

In conclusion, the rutile Ru_x_Ti_1-x_O_2_ nanobelts with exposure of (001) facets were successfully synthesized by a simple sol-gel method chemical route using hydrochloric acid and ethanol as capping and stabilizing agents. The superior photocatalytic activity of rutile nanobelts can be assigned to the band gap energy reduction from 3.2 to 2.55 eV and due to its small size, high surface area and of exposed highly reactive (001) facets. Also, the architecture of the heterostructure with exposure (001) facets favors the separation of photogenerated electron-hole pairs and inhibit the recombination of the electron–hole pairs resulting in the increase of the number of main superoxide and hydroxyl radicals.

A useful application of green technology can be the utilization of the synthesized photocatalysts in the remediation of the environment by decomposition of organic compounds under the sunlight.

## Methods

### Materials

The chemicals used in this work were of analytical reagent. Titanium n-butoxide Ti(OBu)_4_, ruthenium chloride (RuCl_3_xH_2_O), hydrochloric acid (HCl), methylene blue (MB) and NH_4_OH were purchased from Sigma-Aldrich. All solutions were prepared with distilled water.

### Preparation of Ru_x_Ti_1-x_O_2_ nanobelts

The 1D (one-dimension) Ru_x_Ti_1-x_O_2_ nanobelts were synthesized via a sol-gel method described by Nguyen-Phan at al. 20. A typical synthesis implies mixing at room temperature for 30 minutes, of 14 mL of titanium n-butoxide and 14 mL of hydrochloric acid (35 wt.%). Then a desirable amount of RuCl_3_xH_2_O was added into the solution. The mixture was stirred for 12 h at 110 °C. Finally, the resulting precipitate was washed with aqueous 0.1 M NH_4_OH solution (20 ml), and with distilled water. After drying at 70 °C overnight, the products were calcinated in the air at 850 °C for 3 hours. The samples were denoted as Ru_x_Ti_1-x_O_2_, where x represented the nominal doping dosage of ruthenium (x = 0.01, 0.07 and 0.16). The TiO_2_ rutile sample was synthesized by a similar method without adding RuCl_3_xH_2_O precursor and labeled as TiO_2_.

### Characterization

X-Ray diffraction (XRD) of the samples was analyzed at ambient temperature on a Bruker D8 Advance diffractometer using the characteristic Kα radiation of copper at a voltage of 40 kV and a current of 40 mA. XRD patterns were collected in the 2θ range between 5° and 80°.

The UV-vis diffuse reflectance spectra of rutile Ru_x_Ti_1-x_O_2_ were obtained by using a Jasco UV-Vis V-550 spectrophotometer in the wavelength range from 200 to 900 nm with an integrating sphere assembly. The sample was diluted with MgO (ratio 1:6) and then mechanically mixed. The UV-vis absorption was transformed according to the Kubelka Munk function, $F({R}_{\infty })$, for infinite thick samples. The sample surface elements and their oxidation states were analyzed by Thermo Scientific K-Alpha X-ray Photoelectron Spectrometer (XPS) system with Al K-alfa radiation. The Raman spectra of samples were registered using a DXR Raman Microscope from Thermo Scientific. The morphology of the samples was characterized by transmission electron microscope Tecnai™ G2 F20 TWIN Cryo-TEM, FEI Company™, through bright field (BF-TEM) and scanning transmission electron microscopy analyses. The TEM was operated under an acceleration voltage of 200 kV. A small drop of well-dispersed sample, ultrasonicated for 5 minutes, was put on a carbon film copper grid and then, visualized on TEM.

### Photocatalytic experiments

The photocatalytic studies of the rutile Ru_x_Ti_1-x_O_2_ and rutile TiO_2_ composites were evaluated via the degradation of MB under sunlight with a light intensity I = 40000 lx. For photocatalysis analysis, 0,1 g of the photocatalyst was suspended in a beaker with 50 ml aqueous of MB C_0_ = 10^−4^ mol/l. Before exposure to sunlight, the reaction systems were kept in the dark for 30 min to reach absorption-desorption equilibrium between Ru_x_Ti_1-x_O_2_ photocatalysts and MB solution. The absorption intensity of MB was monitoring by using a UV-vis spectrophotometer Jasco UV-vis V-540. At 30 min intervals, 2 ml solution was collected and it was measured absorption at 665 nm (sample is recovered).

The degradation efficiency (DC) of MB can be calculated via the formula:

Degradation efficiency $(DC \% )=\frac{{c}_{o}-c}{{c}_{o}}$, where *C*_0_ *and C* are concentrations of MB initial respectively measured at every 30 min. The photodegradation of MB follows pseudo-first order kinetics.
